# Supplementary material for: Universal classification of twisted, strained and sheared graphene moiré superlattices
Source: Sci Rep. 2016 May 16;6:25670. doi: 10.1038/srep25670 (PMC4867435; doi:10.1038/srep25670)
Supplement: Supplementary Information [file srep25670-s1.pdf]

Supplementary information for: Universal classification of twisted, strained and sheared graphene moiré superlattices

Artaud A.,[1,2,3] Magaud L.,[1,2] Lê Quang T.,[1,3] Guisset V.,[1,2] David P.,[1,2] Chapelier C.,[1,3] and Coraux J.[1,2]

[1] *Univ. Grenoble Alpes, F-38000 Grenoble, France*
[2] *CNRS, Inst NEEL, F-38000 Grenoble, France*
[3] *CEA, INAC-PHELIQS, F-38000 Grenoble, France*

I. FROM COMMENSURABILITY RELATIONS TO STRUCTURAL PARAMETERS

A. Wood's notation

The structural information of a superlattice lies entirely in the commensurability relations. In their most general form (sheared or not), they state as:

$$\begin{pmatrix} \mathbf{a_{m_1}} \\ \mathbf{a_{m_2}} \end{pmatrix} = \begin{pmatrix} i & j \\ k & l \end{pmatrix} \begin{pmatrix} \mathbf{a_{gr_1}} \\ \mathbf{a_{gr_2}} \end{pmatrix} = \begin{pmatrix} m & n \\ q & r \end{pmatrix} \begin{pmatrix} \mathbf{a_{s_1}} \\ \mathbf{a_{s_2}} \end{pmatrix}$$

$$\begin{pmatrix} \mathbf{k_{gr_1}} \\ \mathbf{k_{gr_2}} \end{pmatrix} = \begin{pmatrix} i & k \\ j & l \end{pmatrix} \begin{pmatrix} \mathbf{k_{m_1}} \\ \mathbf{k_{m_2}} \end{pmatrix} \text{ and } \begin{pmatrix} \mathbf{k_{s_1}} \\ \mathbf{k_{s_2}} \end{pmatrix} = \begin{pmatrix} m & q \\ n & r \end{pmatrix} \begin{pmatrix} \mathbf{k_{m_1}} \\ \mathbf{k_{m_2}} \end{pmatrix} \tag{S1}$$

A direct relation between the lattice vectors of graphene and its support is deduced from the above relations as:

$$\begin{pmatrix} \mathbf{a_{gr_1}} \\ \mathbf{a_{gr_2}} \end{pmatrix} = \frac{1}{il - jk} \begin{pmatrix} lm - jq & ln - jr \\ -km + iq & -kn + ir \end{pmatrix} \begin{pmatrix} \mathbf{a_{s_1}} \\ \mathbf{a_{s_2}} \end{pmatrix} = \begin{pmatrix} a & b \\ c & d \end{pmatrix} \begin{pmatrix} \mathbf{a_{s_1}} \\ \mathbf{a_{s_2}} \end{pmatrix} \tag{S2}$$

On the other hand, the (2×2) matrix that links $(\mathbf{a_{gr_1}}, \mathbf{a_{gr_2}})$ and $(\mathbf{a_{s_1}}, \mathbf{a_{s_2}})$ can be written in the formalism of an extended Wood's notation. For a $(p_1 \text{ R}\varphi_1 \times p_2 \text{ R}\varphi_2)$, and assuming a hexagonal support lattice, this matrix rewrites as:

$$\begin{pmatrix} \mathbf{a_{gr_1}} \\ \mathbf{a_{gr_2}} \end{pmatrix} = \begin{pmatrix} p_1 \left(\cos\varphi_1 + \frac{\sin\varphi_1}{\sqrt{3}}\right) & \frac{2p_1}{\sqrt{3}}\sin\varphi_1 \\ -\frac{2p_2}{\sqrt{3}}\sin\varphi_2 & p_2 \left(\cos\varphi_2 - \frac{\sin\varphi_2}{\sqrt{3}}\right) \end{pmatrix} \begin{pmatrix} \mathbf{a_{s_1}} \\ \mathbf{a_{s_2}} \end{pmatrix} \tag{S3}$$

To extract the values of parameters p_1, p_2, φ_1 and φ_2, the matrices from Equations (S2) and (S3) are identified. This gives rise to two non-linear systems of two equations with two unknowns each. It can be solved straightforwardly by considering:

$$\frac{a}{b} = \frac{\sqrt{3}}{2} \frac{1}{\tan\varphi_1} + \frac{1}{2} \quad \Longrightarrow \quad \varphi_1 = \arctan\left(\frac{b\sqrt{3}}{2a - b}\right) \tag{S4}$$

In order to get p_1, the value of φ_1 is inserted into that of b so:

$$b = \frac{2p_1}{\sqrt{3}} \sin\left(\arctan\left(\frac{b\sqrt{3}}{2a - b}\right)\right) \quad \Longrightarrow \quad p_1 = \sqrt{a^2 + b^2 - ab} \tag{S5}$$

Similarly, one can express p_2 and φ_2 as:

$$\varphi_2 = \arctan\left(\frac{c\sqrt{3}}{c - 2d}\right) \tag{S6}$$

$$p_2 = \sqrt{c^2 + d^2 - cd} \tag{S7}$$

Using these four formula, the commensurability relation of any anisotropic superstructure is translated into an extended Wood's notation.

When a structure is isotropic, the commensurability relation simplifies. Instead of eight integer parameters (i, j, k, l, m, n, q, r), only four of them (i, j, m, n) are sufficient. In this case, the (2×2) matrix relating $(\mathbf{a_{gr_1}}, \mathbf{a_{gr_2}})$ and $(\mathbf{a_{s_1}}, \mathbf{a_{s_2}})$ expresses as:

$$\begin{pmatrix} \mathbf{a_{gr_1}} \\ \mathbf{a_{gr_2}} \end{pmatrix} = \frac{1}{i^2 + j^2 - ij} \begin{pmatrix} im - j(m-n) & in - jm \\ jm - in & i(m-n) + jn \end{pmatrix} \begin{pmatrix} \mathbf{a_{s_1}} \\ \mathbf{a_{s_2}} \end{pmatrix} = \begin{pmatrix} a & b \\ -b & a-b \end{pmatrix} \begin{pmatrix} \mathbf{a_{s_1}} \\ \mathbf{a_{s_2}} \end{pmatrix} \tag{S8}$$

This can be translated into a Wood's notation $(p \times p)\mathrm{R}\varphi$ using Equations (S4), (S5), (S6), (S7) with $c = -b$ and $d = a - b$:

$$p = \sqrt{a^2 + b^2 - ab} = \sqrt{\frac{m^2 + n^2 - mn}{i^2 + j^2 - ij}} \tag{S9}$$

$$\varphi = \arctan\left(\frac{b\sqrt{3}}{2a - b}\right) = \arctan\left(\frac{(in - jm)\sqrt{3}}{2(im + jn) - (in + jm)}\right) \tag{S10}$$

B. Strain levels

In order to access the strain levels, the (2×2) matrix that links $(\mathbf{a_{gr_1}}, \mathbf{a_{gr_2}})$ and $(\mathbf{a_{s_1}}, \mathbf{a_{s_2}})$ has to be considered as a plane transformation for example consisting in:

- An isotropic rescaling by a factor p_{iso} corresponding to biaxial strain,
- An anisotropic rescaling by a factor p_{an} in a direction given by an angle θ_1,
- An additional rotation by an angle θ_2.

As a result, one gets:

$$\begin{aligned} \begin{pmatrix} \mathbf{a_{gr_1}} \\ \mathbf{a_{gr_2}} \end{pmatrix} &= \mathrm{P} \begin{pmatrix} \cos\theta_2 & \sin\theta_2 \\ -\sin\theta_2 & \cos\theta_2 \end{pmatrix} \begin{pmatrix} p_{an} & 0 \\ 0 & 1 \end{pmatrix} \begin{pmatrix} \cos\theta_1 & \sin\theta_1 \\ -\sin\theta_1 & \cos\theta_1 \end{pmatrix} \begin{pmatrix} p_{iso} & 0 \\ 0 & p_{iso} \end{pmatrix} \mathrm{P}^{-1} \begin{pmatrix} \mathbf{a_{s_1}} \\ \mathbf{a_{s_2}} \end{pmatrix} \\ &= p_{\mathrm{iso}}\, \mathrm{P} \begin{pmatrix} p_{\mathrm{an}}\cos\theta_1\cos\theta_2 - \sin\theta_1\sin\theta_2 & p_{\mathrm{an}}\sin\theta_1\cos\theta_2 + \cos\theta_1\sin\theta_2 \\ -p_{\mathrm{an}}\cos\theta_1\sin\theta_2 - \sin\theta_1\cos\theta_2 & -p_{\mathrm{an}}\sin\theta_1\sin\theta_2 + \cos\theta_1\cos\theta_2 \end{pmatrix} \mathrm{P}^{-1} \begin{pmatrix} \mathbf{a_{s_1}} \\ \mathbf{a_{s_2}} \end{pmatrix} \end{aligned} \tag{S11}$$

With $\mathrm{P} = \begin{pmatrix} 1 & 0 \\ -1/2 & \sqrt{3}/2 \end{pmatrix}$ the matrix that links a hexagonal to an orthonormal basis.

Identifying Equations (S11) with (S2) leads to a non-linear system of four equations with four unknowns. By linearly combining them, one gets:

$$\begin{cases} \cos(\theta_1 + \theta_2)\, p_{\mathrm{iso}}\, (p_{\mathrm{an}} + 1) = a + d & \text{(S12a)} \\ \sin(\theta_1 + \theta_2)\, p_{\mathrm{iso}}\, (p_{\mathrm{an}} + 1) = \dfrac{-a + 2b - 2c + d}{\sqrt{3}} & \text{(S12b)} \\ \cos(\theta_1 - \theta_2)\, p_{\mathrm{iso}}\, (p_{\mathrm{an}} - 1) = a - b - d & \text{(S12c)} \\ \sin(\theta_1 - \theta_2)\, p_{\mathrm{iso}}\, (p_{\mathrm{an}} - 1) = \dfrac{a + b + 2c - d}{\sqrt{3}} & \text{(S12d)} \end{cases}$$

$\theta_1 + \theta_2$ and $\theta_1 - \theta_2$ can readily be extracted from Equations $\frac{\text{(S12b)}}{\text{(S12a)}}$ and $\frac{\text{(S12d)}}{\text{(S12c)}}$, which are then linearly combined to get:

$$\begin{cases} \tan(2\theta_1) = \sqrt{3}\left(-1 + 2\dfrac{(a^2+c^2+ac)-(b^2+d^2+bd)}{2(a^2+c^2+ac)-(b^2+d^2+bd)-2(ab+cd)-(ad+bc)}\right) & \text{(S13a)} \\ \tan(2\theta_2) = \sqrt{3}\left(1 - 2\dfrac{(a^2+b^2-ab)-(c^2+d^2-cd)}{(a^2+b^2-ab)-2(c^2+d^2-cd)-2(ac+bd)+(ad+bc)}\right) & \text{(S13b)} \end{cases}$$

Moreover, the determinants of the matrices from Equations (S11) and (S2) also have to be equal as:

$$p_{\text{iso}}^2 p_{\text{an}} = ad - bc \tag{S14}$$

This latter equation relates p_{iso} and p_{an} directly. In order to get another relation relating p_{iso} to p_{an}, one can calculate Equations (S12a) × (S12c) + (S12b) × (S12d) and (S12b) × (S12c) − (S12a) × (S12d) as:

$$\begin{cases} p_{\text{iso}}^2\left(p_{\text{an}}^2 - 1\right)\cos(2\theta_2) = \dfrac{2}{3}\left((a^2+b^2-ab)-2(c^2+d^2-cd)+(ad+bc)-2(ac+bd)\right) & \text{(S15a)} \\ p_{\text{iso}}^2\left(p_{\text{an}}^2 - 1\right)\sin(2\theta_2) = \dfrac{-2}{\sqrt{3}}\left((a^2+b^2-ab)-(ad+bc)+2(ac+bd)\right) & \text{(S15b)} \end{cases}$$

And then combine them as Equation $\sqrt{(\text{S15a})^2 + (\text{S15b})^2}$:

$$p_{\text{iso}}^2\left(p_{\text{an}}^2 - 1\right) = \frac{2}{3}\sqrt{\left[a^2+b^2-ab+c^2+d^2-cd+4(ac+bd)-2(ad+bc)\right]^2 + 3\left[a^2+b^2-ab+c^2+d^2-cd\right]^2} \tag{S16}$$

Another relation is obtained using Equation $(\text{S12a})^2 + (\text{S12b})^2$:

$$p_{\text{iso}}^2\left(p_{\text{an}} + 1\right)^2 = \frac{4}{3}\left[(a^2+b^2-ab)+(c^2+d^2-cd)+ac+ad-2bc+bd\right] \tag{S17}$$

Using $p_1 = \sqrt{a^2+b^2-ab}$ and $p_2 = \sqrt{c^2+d^2-cd}$ and $A = 2(ac+bd)-(ad+bc)$, these two latter relations can be rewritten as Equation ½(S17) + ½(S16) − (S14):

$$p_{\text{iso}}^2 p_{\text{an}}^2 = \frac{2(p_1^2+p_2^2)+A}{3}\left(1 + \sqrt{1 + 3\frac{A^2 - 4p_1^2p_2^2}{(2(p_1^2+p_2^2)+A)^2}}\right) \tag{S18}$$

Finally, p_{iso} and p_{an} can be calculated as Equations $\sqrt{(\text{S18}) - (\text{S16})}$ and $\sqrt{\dfrac{(\text{S18})}{(\text{S18}) - (\text{S16})}}$:

$$\begin{cases} p_{\text{iso}} = \sqrt{\dfrac{2(p_1^2+p_2^2)+A}{3}}\sqrt{1 - \sqrt{1 - 3\dfrac{4p_1^2p_2^2 - A^2}{(2(p_1^2+p_2^2)+A)^2}}} & \text{(S19a)} \\ p_{\text{an}} = \dfrac{2(p_1^2+p_2^2)+A}{\sqrt{3(4p_1^2p_2^2 - A^2)}}\left(1 + \sqrt{1 - 3\dfrac{4p_1^2p_2^2 - A^2}{(2(p_1^2+p_2^2)+A)^2}}\right) & \text{(S19b)} \end{cases}$$

Using $B = \dfrac{2(p_1^2+p_2^2)+A}{3}$ and $C = \sqrt{\dfrac{4p_1^2p_2^2 - A^2}{3}}$, this can be written simply as:

$$\begin{cases} p_{\text{iso}} = \sqrt{B - \sqrt{B^2 - C^2}} & \text{(S20a)} \\ p_{\text{an}} = \dfrac{B + \sqrt{B^2 - C^2}}{C} & \text{(S20b)} \end{cases}$$

One can define biaxial and uniaxial strain levels ϵ_{b} and ϵ_{u} using HOPG as a reference. They are related to these geometrical scaling factors p_{iso} and p_{an} as:

$$\begin{cases} \epsilon_{\mathrm{b}} = \dfrac{a_{\mathrm{supp}}}{a_{\mathrm{HOPG}}} p_{\mathrm{iso}} - 1 = \dfrac{a_{\mathrm{supp}}}{a_{\mathrm{HOPG}}} \sqrt{B - \sqrt{B^2 - C^2}} - 1 & \text{(S21a)} \\ \epsilon_{\mathrm{u}} = \dfrac{a_{\mathrm{supp}}}{a_{\mathrm{HOPG}}} p_{\mathrm{iso}} \left(p_{\mathrm{an}} - 1\right) = \dfrac{a_{\mathrm{supp}}}{a_{\mathrm{HOPG}}} \left(\sqrt{B + \sqrt{B^2 - C^2}} - \sqrt{B - \sqrt{B^2 - C^2}} \right) & \text{(S21b)} \end{cases}$$

In other words, graphene is strained anisotropically, and the strain level ranges from $\epsilon = \epsilon_{\mathrm{b}}$ to $\epsilon = \epsilon_{\mathrm{b}} + \epsilon_{\mathrm{u}}$ depending on the in-plane direction.

For an isotropic structure, there cannot be any anisotropic strain, and isotropic strain ϵ is simply given by Equation (S21a) with $c = -b$ and $d = a - b$:

$$\epsilon = \frac{a_{\mathrm{supp}}}{a_{\mathrm{HOPG}}} p - 1 = \frac{a_{\mathrm{supp}}}{a_{\mathrm{HOPG}}} \sqrt{\frac{m^2 + n^2 - mn}{i^2 + j^2 - ij}} - 1 \tag{S22}$$

II. HIGHER ORDER MOIRÉ RELATIONS

In the main text, the moiré could be systematically interpreted using the general relation:

$$N_1 \ \mathbf{k_{m_1}} = \mathbf{k_{gr_1}} - \mathbf{k_{s_1}} \tag{S23}$$

where $N_1 = \sqrt{(i-m)^2 + (j-n)^2 - (i-m)(j-n)}$. This equation has an equivalent counterpart for $\mathbf{k_{m_2}}$. As these relations rely on $\mathbf{k_{gr_1}}$ and $\mathbf{k_{s_1}}$, which are the first graphene and substrate harmonics, they are called 1st order moiré relations. Still, it is possible de define alternative relations using higher harmonics, as performed in Ref. 1, giving rise to so-called 2nd, 3rd and 4th order moiré relations such as:

$$N_1 \ \mathbf{k_{m_1}} = \mathbf{k_{gr_1}} + \mathbf{k_{gr_2}} - 2\mathbf{k_{s_1}} \tag{S24}$$
$$N_1 \ \mathbf{k_{m_1}} = \mathbf{k_{gr_1}} + 2\mathbf{k_{gr_2}} - 3\mathbf{k_{s_1}} \tag{S25}$$
$$N_1 \ \mathbf{k_{m_1}} = \mathbf{k_{gr_1}} + 3\mathbf{k_{gr_2}} - 4\mathbf{k_{s_1}} \tag{S26}$$

It should be noted that for each of these relations, the number of beatings N_1 along $\mathbf{k_{m_1}}$ is different. For instance, the so-called R30 phase of gr/Ir contains $N = 37$ beatings in a 1st order moiré picture, but $N = 1$ beating using the 2nd order moiré relation, which enabled its identification[2].

In the following sections, the equation of the parametrized curves, relating the moiré lattice parameter a_m to both the twist angle φ and the strain level is derived whatever the considered moiré order. Using commensurability constraints, general formulas for the number of beatings are then derived.

A. Parametrized curves

With no commensurability hypothesis and assuming unsheared structures, the moiré superlattice has a D_{6h} symmetry, so $N_1 = N_2 = \sqrt{N}$. Moreover, to relate only graphene and its support, a plane transformation corresponding only to a rotation-scaling is needed:

$$\begin{pmatrix} \mathbf{k_{gr_1}} \\ \mathbf{k_{gr_2}} \end{pmatrix} = \mathrm{P} \begin{pmatrix} p & 0 \\ 0 & p \end{pmatrix} \begin{pmatrix} \cos\varphi & -\sin\varphi \\ \sin\varphi & \cos\varphi \end{pmatrix} \mathrm{P}^{-1} \begin{pmatrix} \mathbf{k_{s_1}} \\ \mathbf{k_{s_2}} \end{pmatrix} \tag{S27}$$

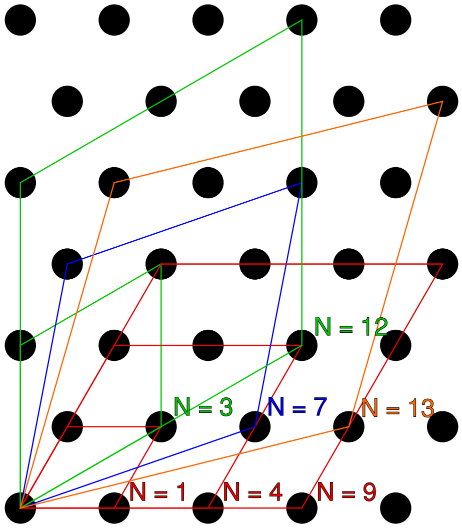

FIG. S1. Scheme enumerating the hexagonal moiré cells for an increasing value of moiré cells N per superstructure unit cell. Each moiré hill is displayed as a black circle.

With $\mathrm{P} = \begin{pmatrix} 1 & 0 \\ -1/2 & \sqrt{3}/2 \end{pmatrix}$ the matrix that links a hexagonal to an orthonormal basis, and $p = \frac{a_{\mathrm{gr}}}{a_{\mathrm{supp}}} = \frac{a_{\mathrm{HOPG}}}{a_{\mathrm{supp}}}(\epsilon - 1)$ defined as the scaling factor.

Using one of Equations (S23), (S24), (S25) or (S26) and Equation (S27), $\mathbf{k_{m_1}}$ is decomposed on the support reciprocal basis $(\mathbf{k_{s_1}}, \mathbf{k_{s_2}})$. In the case of a 1st order moiré, it gives:

$$\sqrt{N}\ \mathbf{k_{m_1}} = \mathbf{k_{gr_1}} - \mathbf{k_{s_1}} \tag{S28}$$

$$= \left(p\ \cos\varphi - \frac{p\sqrt{3}}{3}\sin\varphi - 1\right)\mathbf{k_{s_1}} + \frac{2p\sqrt{3}}{3}\sin\varphi\ \mathbf{k_{s_2}} \tag{S29}$$

From which the moiré period $a_{\mathrm{m}} = \frac{4\pi\sqrt{3}}{3\sqrt{\mathbf{k_{m_1}} \cdot \mathbf{k_{m_1}}}}$ is extracted and expressed as a function of a_{s}, p and φ:

$$1^{\mathrm{st}}\ \text{order} : a_{\mathrm{m}} = a_{\mathrm{s}}\sqrt{\frac{N}{1 - 2p\ \cos\varphi + p^2}} \tag{S30}$$

Similarly, using the alternative relations for so-called higher order moirés, one gets:

$$2^{\mathrm{nd}}\ \text{order} : a_{\mathrm{m}} = a_{\mathrm{s}}\sqrt{\frac{N}{4 - 6p\ \cos\varphi + 2p\sqrt{3}\ \sin\varphi + 3p^2}}$$

$$3^{\mathrm{rd}}\ \text{order} : a_{\mathrm{m}} = a_{\mathrm{s}}\sqrt{\frac{N}{9 - 12p\ \cos\varphi + 6p\sqrt{3}\ \sin\varphi + 7p^2}}$$

$$4^{\mathrm{th}}\ \text{order} : a_{\mathrm{m}} = a_{\mathrm{s}}\sqrt{\frac{N}{16 - 20p\ \cos\varphi + 12p\sqrt{3}\ \sin\varphi + 13p^2}}$$

Following this method, any moiré order can be accounted for. The most general case can then be considered, with a relation such as $\sqrt{N}\ \mathbf{k_{m_1}} = g_1\mathbf{k_{gr_1}} + g_2\mathbf{k_{gr_2}} + s_1\mathbf{k_{s_1}} + s_2\mathbf{k_{s_2}}$. According to this convention, 1st and

2nd orders moirés correspond respectively to $(g_1, g_2, s_1, s_2) = (1, 0, -1, 0)$ and $(g_1, g_2, s_1, s_2) = (1, 1, -2, 0)$. In the general case, the period a_{m} is:

$$a_{\text{m}} = a_{\text{s}} \sqrt{\frac{N}{(s_1^2 + s_2^2 + s_1 s_2) + (2(g_1 s_1 + g_2 s_2) + g_1 s_2 + g_2 s_1)p \cos\varphi + (g_1 s_2 - g_2 s_1)p\sqrt{3} \sin\varphi + (g_1^2 + g_2^2 + g_1 g_2)p^2}} \tag{S31}$$

The integer values taken by N are constrained by the hexagonal lattice through the following Diophantine equation: $a^2 + b^2 - ab = N$, where a and b are integers. A geometrical picture of this problem is given on Fig. S1.

B. Number of beatings

With an additional commensurability hypothesis, the possible values for a_{m} become countable, using the commensurability relation given by the (i, j, m, n) indices, as detailed earlier. Moreover, the link established in the previous section between a_{m} and φ at a given strain level ϵ still applies. Using both, a given commensurate structure of indices (i, j, m, n) can be analysed in terms of any moiré order, and for each order, the number of beatings N can be calculated.

Indeed, the number of beatings N within a moiré cell of any order is simply obtained with:

$$N = \frac{\left|g_1 \mathbf{k_{gr_1}} + g_2 \mathbf{k_{gr_2}} + s_1 \mathbf{k_{s_1}} + s_2 \mathbf{k_{s_2}}\right|^2}{\left|\mathbf{k_{m_1}}\right|^2} \tag{S32}$$

The commensurability relation can be expressed in reciprocal space, as in Equation (S1). It can be seen as a decomposition of $\mathbf{k_{gr_1}}$, $\mathbf{k_{gr_2}}$, $\mathbf{k_{s_1}}$ and $\mathbf{k_{s_2}}$ on the $(\mathbf{k_{m_1}}, \mathbf{k_{m_2}})$ basis using integer values. Injecting this into Equation (S32), the number of beating N of any moiré order given by (g_1, g_2, s_1, s_2) is:

$$\begin{aligned} N = & \left(g_1 i + g_2 j + s_1 m + s_2 n\right)^2 \\ & + \left(g_1 j - g_2(i - j) + s_1 n - s_2(m - n)\right)^2 \\ & - \left(g_1 i + g_2 j + s_1 m + s_2 n\right)\left(g_1 j - g_2(i - j) + s_1 n - s_2(m - n)\right) \end{aligned} \tag{S33}$$

In particular, for the aforementioned 1st, 2nd, 3rd and 4th order moiré superlattices, this results in:

$$\begin{cases} N = (i - m)^2 + (j - n)^2 - (i - m)(j - n) & \text{(S34a)} \\ N = (i + j - 2m)^2 + (2j - 2n - i)^2 - (i + j - 2m)(2j - 2n - i) & \text{(S34b)} \\ N = (i + 2j - 3m)^2 + (3j - 3n - 2i)^2 - (i + 2j - 3m)(3j - 3n - 2i) & \text{(S34c)} \\ N = (i + 3j - 4m)^2 + (4j - 4n - 3i)^2 - (i + 3j - 4m)(4j - 4n - 3i) & \text{(S34d)} \end{cases}$$

For a given structure (i, j, m, n), Equation (S33) can be used to calculate the number of beatings for any moiré order. Nevertheless, from the electronic point of view, the 1st order moiré contribution is the one that dominates in the modulation of the potential the electrons are subjected to. Higher order moirés can be considered as small corrections to this modulation. Equation (S33) gives the number of moiré cells N that a correction of nth order has in the moiré unit cell.

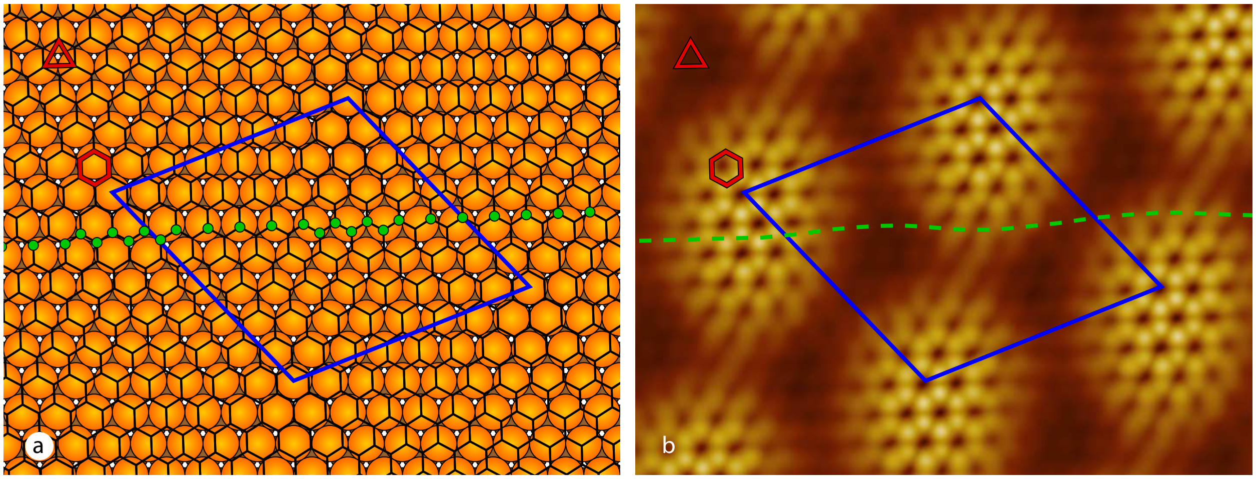

FIG. S2. **DFT simulation of gr/Re**: (**a**) Schematic view of the $(i, j, k, l, m, n, q, r) = (9, 3, -2, 7, 8, 3, -2, 6)$ $N = 1$ moiré superlattice used for DFT calculations. (**b**) Cross-section of the square modulus of the converged wavefunction integrated between E_F and E_F+0.5 eV. On both images, the superlattice unit cell is emphasized with a blue rhombus. In both moiré valleys, only one C out of two is seen in (**b**), contrary to the moiré hill, as indicated by red triangle and hexagon. This alternative appearance of either one C sub-lattice or the other gives rise to an effective C row oscillation (green dots in (**a**), emphasized by a green dotted line in (**b**)).

III. DFT CALCULATIONS OF A SHEARED MOIRÉ SUPERLATTICE OF GR/RE

Density functional calculations were performed in order to provide a qualitative interpretation of the effect of shear strain, in the case of gr/Re, on the observed STM images. The large size of the $N > 1$ beatings moiré superlattice observed experimentally and discussed in the main text is not affordable for DFT calculations, due to a too large number of atoms. A smaller moiré superlattice was hence considered, comprising only one beating. A cross-section in the electronic density map, computed from the relaxed structure, integrated between Fermi level and 0.5 eV above it, is shown on Fig. S2. As discussed in the main text, it reproduces the effective oscillation of the carbon rows and the oval shape of the moiré hills (which are circular in the absence of shear).

IV. POSSIBLE MOIRÉ SUPERLATTICES FOR GRAPHENE ON RHENIUM

Figure S3 displays possible moiré superlattices and observed ones in the case of gr/Re.

V. REPORTED MOIRÉ SUPERLATTICES

Tables S1 and S2 give the list of moiré superlattices that have been explicitly reported for gr/Pt and gr/Ir respectively. As indicated in the main text, these structures cover the full $0 - 30°$ range for $\varphi = (\mathbf{a_s}, \mathbf{a_{gr}})$. Similarly, the strain level $\varepsilon = {}^{(a_{\rm gr} - a_{\rm HOPG})}/_{a_{\rm HOPG}}$ goes up to 4.5%. Given the high number of degrees of freedom epitaxial graphene has to relax strain, it appears unrealistic to consider interpreted superlattices with $\varepsilon \gtrsim 1\%$. Instead, more complex superlattices, with greater moiré periods $a_{\rm m}$, and numerous moiré cells should be considered.

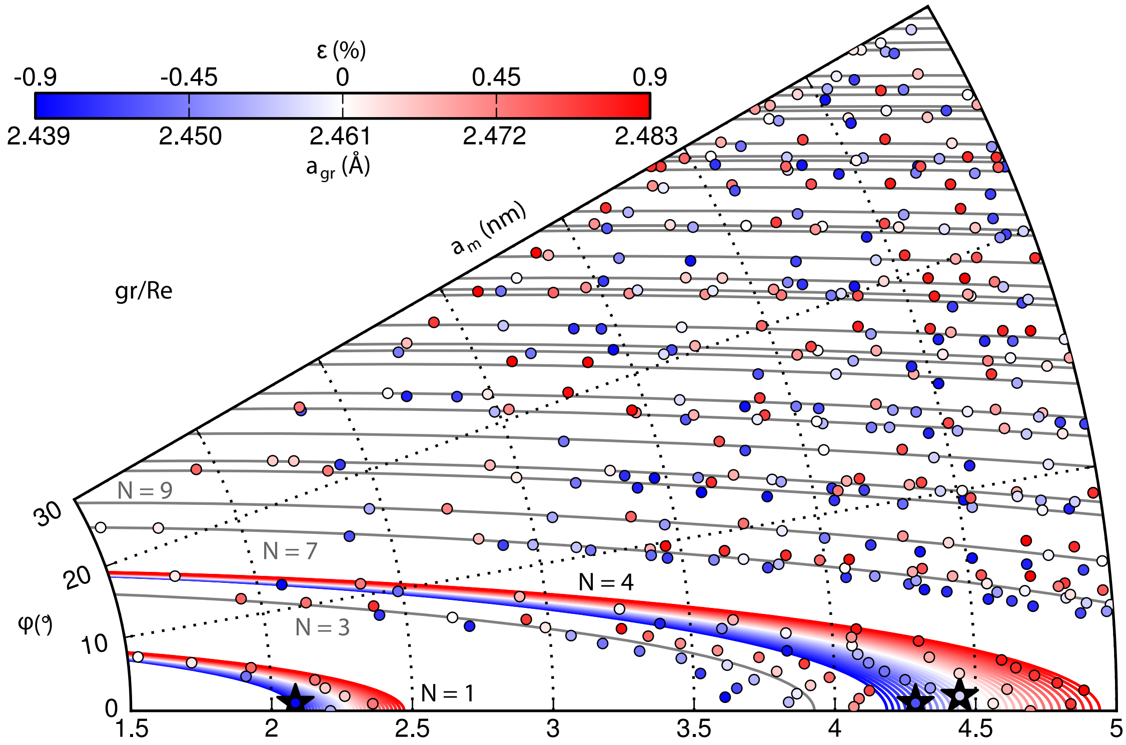

FIG. S3. **Moiré lattice constant a_m versus angle φ between graphene and Re(0001)** ($a_\mathrm{Re} = 2.7609$ Å, see Ref. 3). Each point corresponds to a commensurate superlattice of given a_m and φ, with its colour indicating the strain level of graphene. The full lines indicate the superlattices containing N beatings within the moiré cell. The coloured ones add a strain information, and for clarity are only shown for the $N = 1$ case. Black stars index the sheared structure analysed here.

[1] Zeller, P. & Günther, S. What are the possible moiré patterns of graphene on hexagonally packed surfaces? New J. Phys. **16**, 083028 (2014).

[2] Loginova, E., Nie, S., Thürmer, K., Bartelt, N. C. & McCarty, K. F. Defects of graphene on Ir(111): rotational domains and ridges. Phys. Rev. B **80**, 085430 (2009).

[3] King, H. W. CRC Handbook Of Chemistry And Physics. (eds Lide, D. R.) Ch. 12, 10–12 (CRC press, 74th edition, 1993–1994).

[4] Land, T. A., Michely, T., Behm, R. J., Hemminger, J. C. & Comsa, G. STM investigation of single layer graphite structures produced on Pt (111) by hydrocarbon decomposition. Surf. Sci. **264**, 261–270 (1992).

[5] Merino, P., Švec, M., Pinardi, A. L., Otero, G. & Martín-Gago, J. A. Strain-driven moiré superstructures of epitaxial graphene on transition metal surfaces. ACS Nano **5**, 5627–5634 (2011).

[6] Enachescu, M., Schleef, D., Ogletree, D. F. & Salmeron, M. Integration of point-contact microscopy and atomic-force microscopy: Application to characterization of graphite/Pt (111). Phys. Rev. B **60**, 16913 (1999).

[7] Gao, M. et al. Epitaxial growth and structural property of graphene on Pt(111). Appl. Phys. Lett. **98**, 033101 (2011).

[8] Martínez-Galera, A. J. & Gómez-Rodríguez, J. M. Surface diffusion of simple organic molecules on graphene on Pt(111). J. Phys. Chem. C **115**, 23036–23042 (2011).

[9] Yang, K. et al. Molecule–substrate coupling between metal phthalocyanines and epitaxial graphene grown on Ru(0001) and Pt(111). J. Phys. Chem. C **116**, 14052–14056 (2012).

[10] Sutter, P., Sadowski, J. T. & Sutter, E. Graphene on Pt(111): growth and substrate interaction. Phys. Rev. B **80**, 245411 (2009).

[11] Ugeda, M. M. et al. Point defects on graphene on metals. Phys. Rev. Lett. **107**, 116803 (2011).

gr/Pt	(i,j,m,n)	φ (°)	N	a_{m} (nm)	a_{gr} (Å)	ε (%)	Reference
1	$(9,0,8,0)$	0	1	2.22	2.4661	0.20	4 and 5
2	$(9,1,8,1)$	0.8	1	2.09	2.4516	−0.39	5–7
3	$(10,2,9,2)$	1.3	1	2.27	2.4778	0.67	5
4	$(9,2,8,2)$	1.7	1	2.00	2.4442	−0.69	4 and 5
5	$(10,3,9,3)$	2.1	1	2.20	2.4776	0.67	5
6	$(9,3,8,3)$	2.7	1	1.94	2.4468	−0.59	5 and 6
7	$(9,4,8,4)$	3.7	1	1.92	2.4611	−0.004	5–8
8	$(8,4,7,3)$	4.7	1	1.69	2.4358	−1.03	5 and 9
9	$(8,3,7,2)$	5.7	1	1.73	2.4752	0.57	10
10	$(7,3,6,2)$	6.2	1	1.47	2.4135	−1.94	7
11	$(8,2,7,1)$	6.3	1	1.82	2.5229	2.51	6
12	$(7,2,6,1)$	7.2	1	1.54	2.4735	0.50	5
13	$(6,1,5,0)$	8.9	1	1.39	2.4915	1.23	5 and 6
14	$(5,1,4,0)$	10.9	1	1.11	2.4217	−1.60	5 and 11
15	$(4,0,4,1)$	13.9	1	1.00	2.5008	1.61	5, 7, and 12
16	$(7,0,7,2)$	16.1	4	1.73	2.4752	0.57	5
17	$(3,0,3,1)$	19.1	1	0.73	2.4468	−0.59	5–11
18	$(8,5,7,2)$	22.1	7	1.73	2.4752	0.57	5
19	$(5,0,5,2)$	23.4	4	1.21	2.4187	−1.73	6 and 13
20	$(5,2,4,0)$	23.4	3	1.11	2.5460	3.45	6
21	$(2,0,2,1)$	30	1	0.48	2.4027	−2.38	4, 5, and 7

TABLE S1. **Noticeable gr/Pt moiré superlattices** Each considered superlattice is labelled with its (i,j,m,n) integers, and the corresponding graphene-substrate angle (φ), graphene and moiré lattice constants (a_{gr} and a_{m}), and biaxial strain (ε) are indicated. Indices correspond to those appearing in Fig. 7b of main text.

gr/Ir	(i,j,m,n)	φ (°)	N	a_{m} (nm)	a_{gr} (Å)	ε (%)	Reference
1	$(10,0,9,0)$	0	1	2.44	2.4432	−0.73	14 and 15
2	$(11,0,10,0)$	0	1	2.71	2.4679	0.27	16
	$(21,0,19,0)$	0	4	5.16	2.4562	−0.20	16
3	$(11,4,10,4)$	2.4	1	2.37	2.4541	−0.29	17
4	$(4,0,4,1)$	13.9	1	0.98	2.4470	−0.58	2, 14, and 15
5	$(7,3,6,1)$	16.3	1	1.51	2.4849	0.96	14
6	$(13,1,13,5)$	18.5	16	3.08	2.4607	−0.02	2
7	$(3,0,3,1)$	19.1	1	0.72	2.3941	−2.73	14 and 15
8	$(5,2,4,0)$	23.4	1	1.09	2.4912	1.22	14
9	$(14,9,12,2)$	29.6	37	3.02	2.4601	−0.04	2
10	$(2,0,2,1)$	30	1	0.47	2.3510	−4.48	14

TABLE S2. **Noticeable gr/Ir moiré superlattices** Each considered superlattice is labelled with its (i,j,m,n) integers, and the corresponding graphene-substrate angle (φ), graphene and moiré lattice constants (a_{gr} and a_{m}), and biaxial strain (ε) are indicated. Indices correspond to those appearing in Fig. 7a of main text.

[12] Ueta, H. et al. Highly oriented monolayer graphite formation on Pt(111) by a supersonic methane beam. Surf. Sci. **560**, 183–190 (2004).

[13] Grandthyll, S. et al. Epitaxial growth of graphene on transition metal surfaces: chemical vapor deposition versus liquid phase deposition. J. Phys. Condens. Matter **24**, 314204 (2012).

[14] Meng, L. et al. Multi-oriented moiré superstructures of graphene on Ir(111): experimental observations and theoretical models. J. Phys. Condens. Matter **24**, 314214 (2012).

[15] Zeller, P., Dänhardt, S., Gsell, S., Schreck, M. & Wintterlin, J. Scalable synthesis of graphene on single crystal Ir(111) films. Surf. Sci. **606**, 1475–1480 (2012).

[16] Blanc, N. et al. Local deformations and incommensurability of high-quality epitaxial graphene on a weakly interacting transition metal. Phys. Rev. B **86**, 235439 (2012).

[17] Jean, F. et al. Effect of preparation on the commensurabilities and thermal expansion of graphene on Ir(111) between 10 and 1300 K. Phys. Rev. B **88**, 165406 (2013).
